# Some Ideas on Alteratives

**Published:** 1898-12

**Authors:** H. G. Reemsnyder

**Affiliations:** Ephrata, Pa.


### SOME IDEAS ON ALTERATIVES.

BY H. G. REEMSNYDER, M.D., EPHRATA, PA.

The word "alterative" is construed by a great many medical writers as a term that can be applied to any drug or remedy of whose therapeutics not much is known, and less written. Consequently our materia medica is full of so-called alteratives, many of which are seldom used and possess an action far different from that ascribed to them, but for want of a better term are thus denominated.

According to Brunton an alterative is a drug that improves the general condition of the body, stimulates nutrition, and regulates the waste elimination without exerting any perceptible action on individual organs.

This clearly defines the therapeutic action of those drugs called alteratives, a study of which is always interesting, since it is to them that much of the success in the treatment of chronic diseases is due.

The knowledge of the fact that a great many chronic diseases and conditions are amenable to treatment with benefit by alteratives is not by any means confined to the profession of medicine, but it has reached the laity and has spread through all the length and breadth of the land, as is evidenced by the general use of what are known as "blood purifiers," some of which have an enormous sale.

It is, therefore, essential to the physician to be acquainted with the drugs which are thus employed, as there is scarcely a condition of a chronic nature in which a tonic alterative will not be of benefit. Every physician must have noted that there are always a certain number of patients among his clientele who may be called "regulars," who require a course of medical treatment about once every month or two, in order to preserve their peace of mind, if not that of their body; who come with a train of symptoms, as varied and as remarkable as they themselves are, and which are withal so vague and incomplete that no earthly physician can diagnose their troubles, who, nevertheless, will be greatly benefited by an alterative which will improve the nutrition, eliminate waste, and not affect any individual organ.

Without doubt our best alteratives are to be found in the vegetable kingdom, and these have the advantage of being free from harm in prolonged administration, which can not always be said of the indiscriminate use of such powerful mineral substances as mercury or arsenic. Another advantage can be attained in the use of vegetable remedies in the preparation of the green drug, upon which point I believe almost all authorities agree.

There has been, however, on the part of our manufacturing chemists somewhat of a tendency to ignore the demand for green drug extracts, so that it has become almost impossible to obtain reliable extracts which are made from the green drug. However, we have one preparation of this character that experience has proved reliable, and which for certain therapeutic action is unsurpassed. I have reference to that known as iodia, which is composed of the active principles obtained from the green roots of stillingia, helonias, saxifraga, and menisperum, to which are added ferri phosphatum and potassium iodid. In theory this preparation presents an ideal formula, while in practice it produces certain beneficial results.

In addition to those cases mentioned as being susceptible to marked benefit by the use of an alterative such as here indicated, this preparation has another and perhaps more important action, that of certainly curing syphilis. I have had ample opportunity to test this remedy in cases of syphilis in all stages, and I am in a position to positively assert that in this disease we can find nothing which gives more promising effects, and which allows us to give more encouragement to the unfortunate victims.

I report a few cases that have come under my care a year or more ago, were treated with iodia, and have remained free from the disease to the present time:

Case 1. Male; age, fourty-four; married; had contracted syphil s about three years before consulting me. He gave a clear history of primary sore, followed by the usual symptoms, and was treated by another physician at that time. He had possession of the prescriptions which he had been using, and they were shown me. Large doses of corrosive sublimate had been prescribed, with arsenic iodid and sarsaparilla. He seemed to improve rapidly, and after a time discontinued treatment. He consulted me for relief from a bursa of the right elbow, which he had been treating with external applications, without relief, of course. In inquiring into his history I asked him whether he ever had syphilis. "Three years ago," he replied, "but that's all right now." I told him plainly that his present trouble undoubtedly was caused by the disease which he had contracted at that time, and that doubtless other signs of the disease could be found. Inquiry further revealed dull pains over the region of the liver, much dull headache, and a peculiar scaly condition of the nails.

I promptly told him that he would require active and persistent treatment to overcome his trouble; explained to him that there was internal indication of syphilis, which would sooner or later be the cause of much mischief if not corrected. With the understanding that he would continue six months at least, I put him on iodia, one teaspoonful three times a day, gradually increasing to one and a half teaspoonfuls. I furnished him with a month's supply, and did not see him until some thirty days after his first visit, when he returned to my office. The bursa had almost entirely disappeared, and he said he was feeling very much better "all through" since he had been taking the medicine. I gave him another month's supply and did not see him again until the medicine had been taken. No vestige of the bursa remained, and as he was feeling very well, it was with the greatest difficulty that I persuaded him of the necessity for continuing the treatment. He, however, did as I requested, and at the end of six months I allowed him to cease treatment and await developments. In an interview with him a few weeks ago, eleven months after treatment was stopped, he reported himself entirely free from anything which could be ascribed to syphilis.

Case 2. Female; age, thirty-one; occupation, servant. Consulted me with the following history: Severe dysmenorrhea, itching sensation all over the body, dull headaches most of the time, leucorrhea occasionally, and a general feeling of malaise and despondency. Told me that another physician had treated her for syphilis, which she had contracted several years before, at the age of twenty-six, and she was intelligent enough to know the virulence of this disease, and ascribe her ailments to it. Treatment with iodia gave marked benefit, and in due time effected a cure, the dysmenorrhea ceasing to be so severe at her first menstruation following treatment, with a total relief lasting over a year, to the present time.

Cases of this kind could be multiplied, but rough outlines are only given to acquaint the reader who may not have given this remedy a trial of its good results. I have found it especially valuable in cases requiring a uterine tonic and general tonic. Those stubborn cases of leucorrhea, ovarian pains, bearing down sensations, etc., will be found, in most cases, where there is no organic trouble, to respond to this treatment. In certain skin diseases, where there is a tendency to scaly formations, itching, or persistent ulcers, with suitable local treatment the remedy will be found a most valuable adjunct.—*Medical and Surgical Reporter, Jan. 16, 1897.*

